# Supplementary material for: Test-retest reliability of multi-metabolite edited MRS at 3T using PRESS and sLASER
Source: bioRxiv. 2025 Jun 11:2025.06.07.657685. Preprint. [Version 1] doi: 10.1101/2025.06.07.657685 (PMC12259001; doi:10.1101/2025.06.07.657685)
Supplement: 1 [file NIHPP2025.06.07.657685v1-supplement-1.pdf]

bioRxiv preprint doi: https://doi.org/10.1101/2025.06.07.657685; this version posted June 11, 2025. The copyright holder for this preprint (which was not certified by peer review) is the author/funder, who has granted bioRxiv a license to display the preprint in perpetuity. It is made available under aCC-BY 4.0 International license.

Table S1. MRSinMRS checklist.

**1. Hardware**	
a. Field strength [T]	3T
b. Manufacturer	GE
c. Model (software version if available)	Discovery MR750 (DV26.0_R01_1725.a)
d. RF coils: nuclei (transmit/ receive), number of channels, type, body part	^{1}H 32-channel phased-array head coil for receive; body coil for transmit
e. Additional hardware	n/a
**2. Acquisition**	
a. Pulse sequence	PRESS- and sLASER-localized HERMES and HERCULES
b. Volume of Interest (VOI) locations	Medial parietal lobe
c. Nominal VOI size	$30 \times 30 \times 30$ mm^3
d. Repetition Time (TR), Echo Time (TE)	TR = 2000 ms; TE = 82 ms
e. Total number of Excitations or acquisitions per spectrum<br>In time series for kinetic studies<br>i. Number of Averaged spectra (NA) per time-point<br>ii. Averaging method (e.g., block-wise or moving average)<br>iii. Total number of spectra (acquired / in time-series)	224
f. Additional sequence parameters (Spectral width in Hz, number of spectral points, frequency offsets)<br>If STEAM: Mixing Time (TM)<br>If MRSI: 2D or 3D, FOV in all directions, matrix size, acceleration factors, sampling method	Spectral width: 5000 Hz<br>Spectral points: 4096
g. Water Suppression Method	CHESS for PRESS-localized acquisitions;<br>VAPOR for sLASER-localized acquisitions

bioRxiv preprint doi: https://doi.org/10.1101/2025.06.07.657685; this version posted June 11, 2025. The copyright holder for this preprint (which was not certified by peer review) is the author/funder, who has granted bioRxiv a license to display the preprint in perpetuity. It is made available under aCC-BY 4.0 International license.

h. Shimming Method, reference peak, and thresholds for "acceptance of shim" chosen	Double-echo GRE
i. Triggering or motion correction method (Respiratory, peripheral, cardiac triggering, incl. device used and delays)	n/a
**3. Data analysis methods and outputs**	
a. Analysis software	Osprey (v2.5.0); R (v4.4.0)
b. Processing steps deviating from quoted reference or product	RF coil combination performed using generalized least squares (An et al., 2013, doi:10.1002/jmri.23941)<br>Customized GE basis sets created for TE = 82 ms PRESS and sLASER multi-metabolite spectral editing using MRSCloud (Hui et al., 2022, doi:10.1002/mrm.29370)
c. Output measure<br>(e.g., absolute concentration, institutional units, ratio)<br>Processing steps deviating from quoted reference or product	Water-referenced metabolite levels (no correction for GM, WM, or CSF)
d. Quantification references and assumptions, fitting model assumptions	Unsuppressed water used as a reference; model assumptions were as set by Osprey by default
**4. Data Quality**	
a. Reported variables<br>(SNR, Linewidth (with reference peaks))	Creatine SNR; unsuppressed water linewidth
b. Data exclusion criteria	Visual inspection of spectra; multivariate outlier exclusion using the Mahalanobis-minimum covariance determinant distance (Leys et al., 2018, doi:10.1016/j.jesp.2017.09.011)
c. Quality measures of postprocessing Model fitting (e.g., CRLB, goodness of fit, SD of residual)	Fit error of the SUM, DIFF1, and DIFF2 Hadamard-combined spectra, calculated as the sum of squares of residuals normalized to the

bioRxiv preprint doi: https://doi.org/10.1101/2025.06.07.657685; this version posted June 11, 2025. The copyright holder for this preprint (which was not certified by peer review) is the author/funder, who has granted bioRxiv a license to display the preprint in perpetuity. It is made available under aCC-BY 4.0 International license.

	square of the noise amplitude and multiplied by the number of points in the residual
d. Sample Spectrum	Provided in the main text of the article
